# Editorial

**Published:** 1890-01

**Authors:** 


### Editorial.

### SALUTATORY.

Having undertaken editorial management of THE SOUTHERN MEDICAL RECORD, we must acknowledge that we enter upon the discharge of our duties with misgivings natural enough with those engaging in new fields of labor. We are a working, not a writing people, therefore the scarcity of medical statistics in the South. This places us at disadvantage with our Northern brethren, who industriously gather, compile and publish everything of consequence that occurs among them. We have not been so doing, notwithstanding our splendid opportunities. *The main object of our effort* is to create more interest and enthusiasm among Southern physicians and encourage them to greater diligence in recording their triumphs and their failures. We will endeavor to retrieve what is possible of the *experience* and *knowledge* of our seniors, now only written on the tablets of memory, ready at any moment to be lost to science and humanity, as has been the case thousands of times in the past.

If we can induce the young men to contribute to our medical literature whatever of interest may occur in their practice, we will not only be highly gratified, but will claim an humble place among the votaries of science and the benefactors of the race.

We declare express allegiance to *honest* and *legitimate medicine*, and, when necessary, will defend the *same* against the aspersions of all enemies.

We will do what we can to preserve harmony in the ranks of the profession, and encourage every undertaking having for its object the good of the profession and of the people.

We will advocate prison reforms, and also houses of correc- tion for those of tender years who may fall in the ways of vice and ruin.

We will urge the establishment of inebriate asylums to suppress and prevent crime and reclaim and restore to society such unfortunates as may be possibly cured.

A. W. GRIGGS, M. D.

WM. PERRIN NICOLSON, M. D.

FRANK O. STOCKTON, M. D.

---

### VALEDICTORY.

The undersigned, heretofore the Editors and Proprietors of THE SOUTHERN MEDICAL RECORD, have now to announce to their numerous readers and friends that they have sold their interest in the RECORD to Dr. D. H. HOWELL, of Atlanta, Ga., who is now the proprietor, and will hereafter publish the journal, commencing with the January number, 1890.

All amounts due the journal up to the close of the present year, or to the first of January, 1890, are due to Dr. Word, the present business manager.

All unexpired subscriptions and contracts will be carried out by the new proprietor, and all dues accruing from and after the 1st day of January, 1890, belong to Dr. Howell and he is authorized to collect the same.

Our readers will, therefore, please remember to remit all amounts due or to become due on the 31st of December, 1889, to Dr. R. C. Word, who will give attention to closing up the old business.

We take leave of our old friends and patrons with a feeling of regret and sadness, of which we have no words adequate to give expression.

Their names, many of them upon our list for a period of twenty years, are to our minds as friends true and beloved, and will ever be cherished in our memories and our hearts.

To our exchanges, too, we feel a warm degree of kindness, and express our high appreciation of the aid derived from them, and our warmest thanks for courtesies extended. They are requested to continue to exchange with the new proprietor.

The step thus taken had become a necessity to each of us by reason of advancing years, and of constantly increasing duties too onerous to be continued. It were better for us, our readers, and for the interest of THE RECORD, that new, young, and more active hands assume the arduous duties which the progress of the times, and the accumulating details of the journal business, demand.

This is made the more necessary by our connection with the Southern Medical College, which has assumed proportions so large as will hereafter demand much time and labor at our hands. While all this is true, we would not be understood as wholly done with the pen, for we hope to find time for an occasional contribution to THE RECORD, as we feel it the duty of every true friend of the profession to do something for the advancement of Medical Literature, and the progress of Medical Science in his section.

We bespeak for THE RECORD the continued patronage and support of all its old friends, and the profession at large.

We trust that all our readers will continue to take the journal, and that they will give Dr. Howell their cordial co-operation and support.

Surely our home journals, of which THE RECORD stands among the first, should be sustained by the physicians in this section.

So mote it be—adieu!

T. S. POWELL, M. D.
R. C. WORD, M. D.

---

The undersigned begs leave to state that the SOUTHERN MEDICAL RECORD has passed into his hands, as proprietor, and that the entire management of its finances will devolve upon him. Therefore, all contracts for advertisements must be made with him, and every business matter referred to him for agreement and settlement. The strictest business principles will be practiced, and I hope to be able to give satisfaction to the most fastidious. I make the appeal to all Southern doctors to write short papers, and to report all of their interesting cases. It is a duty they owe the profession at large. That is the reason our Northern brethren have such a big reputation, for they write up all of their interesting cases for their journals. It is not because they have more interesting cases than we do, for that is impossible; but they are willing to write their experiences for the benefit of the profession at large. This is just what I want our Southern doctors to do for this journal. With their co-operation, and my able editorial staff, I will give them a journal equal to the best that is published in the United States. I also appeal to you to subscribe for my journal, and by so doing help me give them a first-class journal. While I make this special appeal to my Southern colleagues, I do not mean to exclude my Northern brethren, but will be glad to have contributions from all.

I have also enlarged THE RECORD to forty-eight pages of reading matter. and come out in a new dress.

I have secured able foreign correspondents, besides our reguiar Northern correspondents, who will contribute interesting monthly letters.

All communications should be addressed to me at the office of the ROCORD, No. 29 N. Forsyth street, Atlanta Ga.

DAN. H. HOWELL, M. D., Business Manager.

### THE NEW YEAR.

The beginning of the new year inaugurates changes in the management of THE RECORD which have been elsewhere noted in this issue. We come, not as a new departure, but as an old and established journal under improved conditions, with increased facilities, and with earnest desire to make it a true exponent of the medical thought of the south.

To tell the old friends and new, we would call attention to the mutual dependence upon each other of the practitioner and his journal, and to bespeak that assistance and co-operation which can alone perfect our usefulness, and assist us in placing this journal upon the plane which we desire.

There are few men in active practice who cannot do something towards advancing the literature of medicine, and adding the grand total of facts from which deductions must be drawn upon, the result of which the old tenets must fall and the new arise, or the new being tried, their true status determined. It is such intelligent co-operation that we ask for at the hands of the profession of the south.

The history of medicine in this country shows abundant evidence of the impress of Southern minds on the advancement of its highest interests, and in order that this influence shall be still more marked, it is essential that we should devote more attention to society and journal work. Home journals should receive the unstinted support of home doctors and it shall be our highest aim to deserve and command this. In all that tends to advance medical science, and to elevate its dignity, we shall ever be ready to give our warmest support.

Having no end to subserve other than keeping abreast of current medical thought, and furthering the highest aims of the profession, we come to the old friends of THE RECORD with promises of renewed energies in promoting its usefulness, and to the profession at large with the hope, that by holding up a mirror of the latest developments in the medical world, we shall make it a necessity. Laboring with this end in view we feel that our good resolutions for the new year will bear fruit for them and for us.

---

IN the New York *Medical Record* of December 7th, an apropos editorial will be found under the title of "Doctor." It really is time that some distinction was made between those who, simply because they sell tooth-brushes, soaps, etc., over a drug counter, or trim the vegetable products of your toes, yet are known by that magic term, "doctor," or "doc.," and those to whom the title rightly belongs.

---

THE *Annals of Surgery* has now entered upon its sixth year of publication. Much praise is due both to the home and foreign editors for the high literary standard sustained. This is the only journal published anywhere in the English language devoted exclusively to scientific surgery and which does not seek popularity by giving minor surgery, but rather bringing the reader up to the highest literary and practical attainments in surgery, nor does it in the least degree cater to advertisers. The numbers are profusely illustrated with fine engravings and diagrams, elucidating the text. It is well worthy the patronage of all members of the profession who do any surgery. $5.00 per year. Sample copies 50 cents. J. H. Chambers & Co., St. Louis, Mo., are the publishers.

